# Supplementary material for: DMRforPairs: identifying Differentially Methylated Regions between unique samples using array based methylation profiles
Source: BMC Bioinformatics. 2014 May 15;15:141. doi: 10.1186/1471-2105-15-141 (PMC4046028; doi:10.1186/1471-2105-15-141)
Supplement: Additional file 2 — DMRforPairs output for the comparison of A431-MCF7 and NA17018-NA17105. Please start from the HTML files in each folder. Available via the BMC Bioinformatics website. [file 1471-2105-15-141-S2.zip › 1394847754114233_MOESM2_ESM/A431_MCF7/figures/37496.pdf]

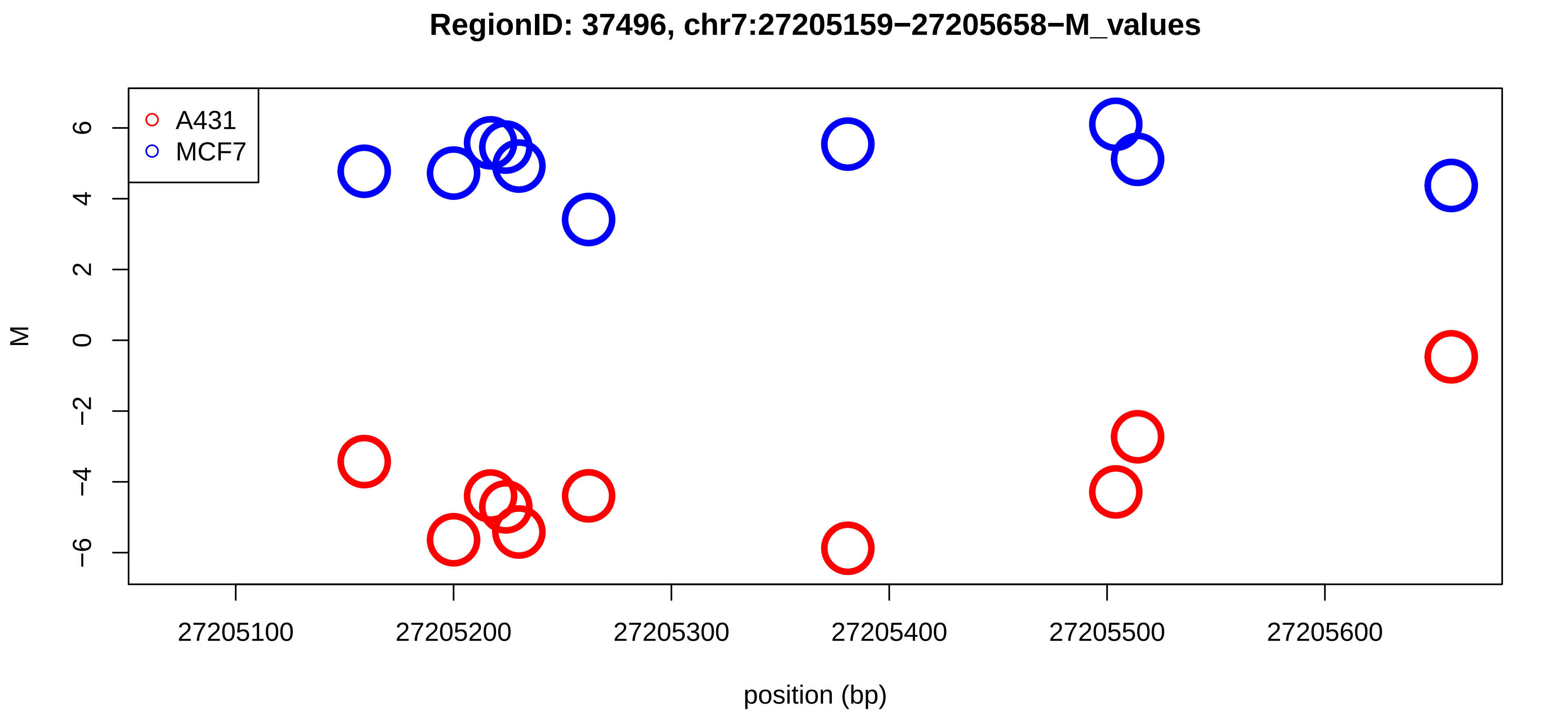

RegionID: 37496, chr7:27205159−27205658−M_values
A431
MCF7
M
6
4
2
0
−2
−4
−6
27205100
27205200
27205300
27205400
27205500
27205600
position (bp)

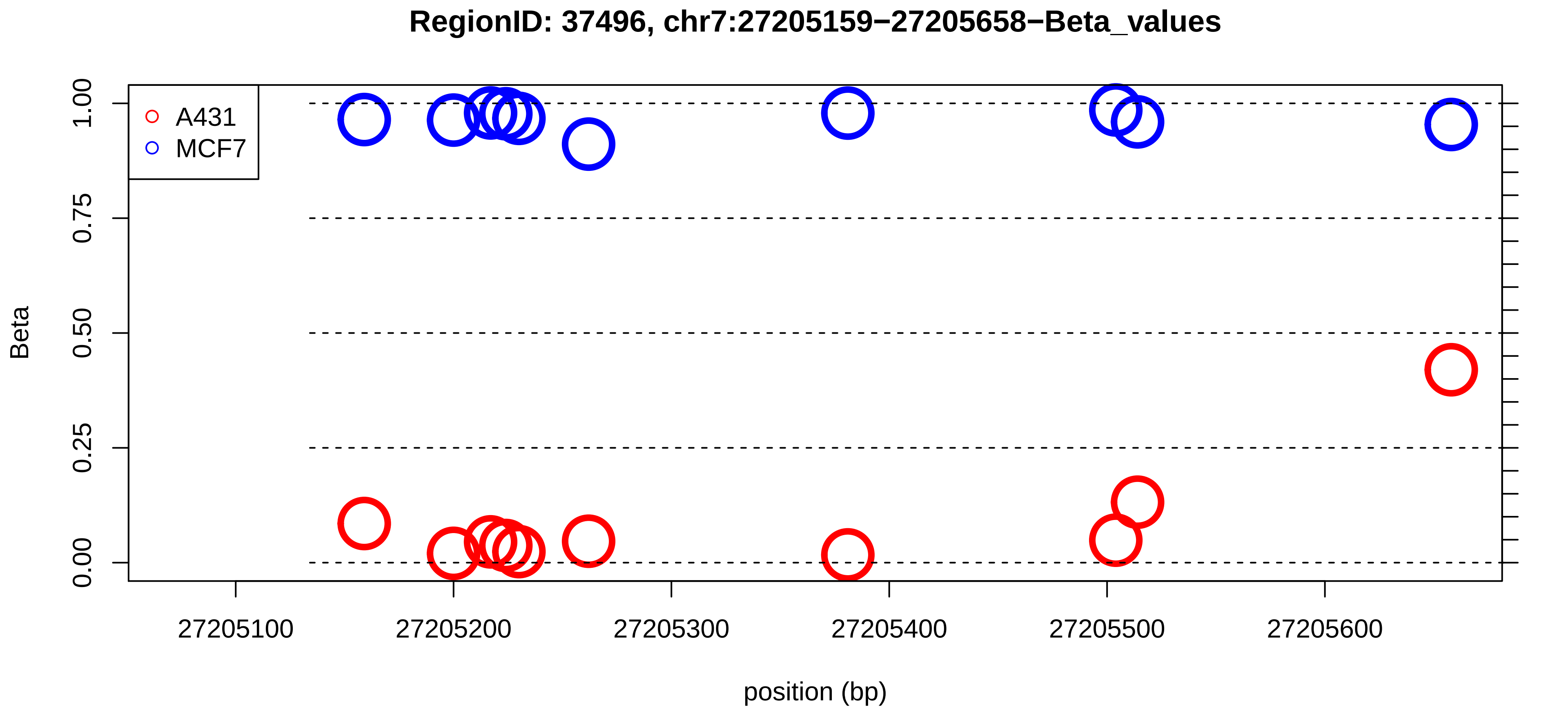

RegionID: 37496, chr7:27205159−27205658−Beta_values
A431
MCF7
Beta
1.00
0.75
0.50
0.25
0.00
27205100
27205200
27205300
27205400
27205500
27205600
position (bp)

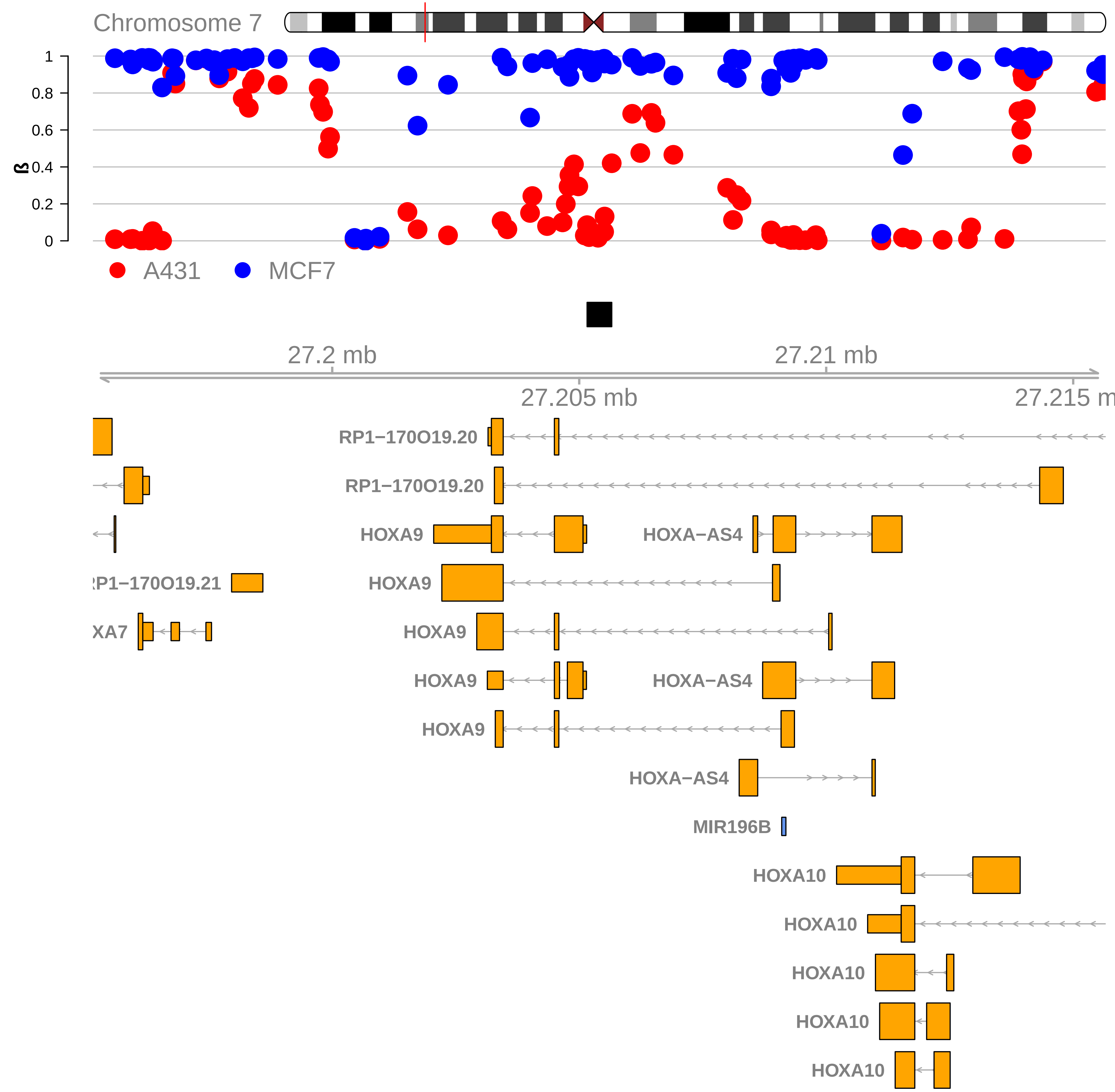

Chromosome 7
1
0.8
0.6
ß 0.4
0.2
0
A431
MCF7
27.2 mb
27.21 mb
27.205 mb
27.215 m
RP1−170O19.20
RP1−170O19.20
HOXA9
HOXA−AS4
RP1−170O19.21
HOXA9
XA7
HOXA9
HOXA9
HOXA−AS4
HOXA9
HOXA−AS4
MIR196B
HOXA10
HOXA10
HOXA10
HOXA10
HOXA10
